# Sphingosine Kinases and Sphingosine 1-Phosphate Receptors: Signaling and Actions in the Cardiovascular System

**DOI:** 10.3389/fphar.2017.00556

**Published:** 2017-08-23

**Authors:** Alessandro Cannavo, Daniela Liccardo, Klara Komici, Graziamaria Corbi, Claudio de Lucia, Grazia D. Femminella, Andrea Elia, Leonardo Bencivenga, Nicola Ferrara, Walter J. Koch, Nazareno Paolocci, Giuseppe Rengo

**Affiliations:** ^1^Lewis Katz School of Medicine, Center for Translational Medicine, Temple University, Philadelphia PA, United States; ^2^Department of Translational Medical Sciences, University of Naples Federico II Naples, Italy; ^3^Department of Medicine and Health Science, University of Molise Campobasso, Italy; ^4^Imperial College London London, United Kingdom; ^5^Istituti Clinici Scientifici Maugeri SpA Società Benefit, Telese Terme Institute (BN) Telese, Italy; ^6^Division of Cardiology, Johns Hopkins University Medical Institutions, Baltimore MD, United States; ^7^Department of Experimental Medicine, University of Perugia Perugia, Italy

**Keywords:** sphingosine 1-phosphate, G protein-coupled receptors, sphingosine kinase, fingolimod, cardiovascular, heart failure, gene-therapy

## Abstract

The sphingosine kinases 1 and 2 (SphK1 and 2) catalyze the phosphorylation of the lipid, sphingosine, generating the signal transmitter, sphingosine 1-phosphate (S1P). The activation of such kinases and the subsequent S1P generation and secretion in the blood serum of mammals represent a major checkpoint in many cellular signaling cascades. In fact, activating the SphK/S1P system is critical for cell motility and proliferation, cytoskeletal organization, cell growth, survival, and response to stress. In the cardiovascular system, the physiological effects of S1P intervene through the binding and activation of a family of five highly selective G protein-coupled receptors, called S1PR_1-5_. Importantly, SphK/S1P signal is present on both vascular and myocardial cells. S1P is a well-recognized survival factor in many tissues. Therefore, it is not surprising that the last two decades have seen a flourishing of interest and investigative efforts directed to obtain additional mechanistic insights into the signaling, as well as the biological activity of this phospholipid, and of its receptors, especially in the cardiovascular system. Here, we will provide an up-to-date account on the structure and function of sphingosine kinases, discussing the generation, release, and function of S1P. Keeping the bull’s eye on the cardiovascular system, we will review the structure and signaling cascades and biological actions emanating from the stimulation of different S1P receptors. We will end this article with a summary of the most recent, experimental and clinical observations targeting S1PRs and SphKs as possible new therapeutic avenues for cardiovascular disorders, such as heart failure.

## Introduction

Sphingolipids are ubiquitous components of the eukaryotic cell membrane that play important roles in the regulation of many cellular processes (Ghosh et al., 1990; Zhang et al., 1991). Among these molecules, sphingosine 1-phosphate (S1P) is a bioactive lipid with a variety of physiological roles across a broad range of organisms (Strub et al., 2010). S1P is produced by the phosphorylation of sphingosine, a reaction catalyzed by an enzyme, sphingosine kinase (SphK), present in two isoforms, SphK1 and 2. S1P degradation involves a cleavage by S1P lyase (SPL) (Imamura et al., 2001). In general, when phosphorylated, this lipid is secreted in the plasma, mainly by red blood cells, platelets, fibroblasts, and vascular endothelial cells (ECs, Tani et al., 2005; Kacimi et al., 2007; Pappu et al., 2007; Venkataraman et al., 2008; Gellings Lowe et al., 2009). In addition, extracellular SphK1 released from these cells may also contribute to S1P synthesis (Venkataraman et al., 2006).

The activation of SphK1 and 2, and the consequent S1P generation/secretion have a crucial role in many cellular signaling cascades and pathological processes. In particular, their role has been widely studied in angiogenesis, in cancer development/progression and in immune and inflammatory responses (Karliner, 2009). However, the SphK/S1P axis has received a special attention from cardiovascular scientists because implicated in the cardiovascular system development and functioning. The latter encompasses the modulation of heart rate, cardiac contractility, and vascular tone (Peters and Alewijnse, 2007). All these effects are mediated by the binding to specific G protein-coupled receptors (GPCRs), called S1PRs (Hla et al., 2001; Chun et al., 2002; Spiegel and Milstien, 2003; Usui et al., 2004). Since an increasing body of experimental evidence supports the notion that activating the SphK/S1P-S1PR system protects both the heart and the vasculature, several synthetic S1P analogs have been previously designed (Pelletier and Hafler, 2012). Of note, one of them, Fingolimod, or FTY720 (a Novartis proprietary compound) is currently approved and used in clinical practice to treat neurological degenerative disorders, such as multiple sclerosis (Pelletier and Hafler, 2012).

Given the current availability of compounds, such as Fingolimod, it is now more feasible, and should be even more attractive, to test the impact of agonists of the SphK/S1P-S1PR axis in the context of clinically relevant cardiovascular disorders, as in the recent case of a study that demonstrated an increase in myocardial salvage and a decrease in adverse post-infarction remodeling with Fingolimod in a porcine model of ischemia-reperfusion injury (Santos-Gallego et al., 2016).

Here, we will review the mechanisms by which SphKs modulate S1P generation and secretion in different cardiovascular compartments. Then, we will focus on the pathophysiological role exerted by SphK/S1P-S1PRs axis in the circulatory system. Finally, we will describe how S1PRs agonism and antagonism can improve outcome in cardiac disease states, such as post-ischemic heart failure (HF).

## Structure and Function of Sphingosine Kinases

### Structure of SphKs

The SphKs are members of a family of enzymes that includes the diacylglycerol (DAG) and the ceramide kinases, all of which are able to generate bioactive lipids (Wattenberg et al., 2006). Currently, two isoforms (SphK1 and 2) have been cloned and characterized, and the genes encoding for these two enzymes are localized on different chromosomes—sphk1 gene is on chromosome 17, whereas sphk2 gene is on chromosome 19—and encode for several splicing variants (Imamura et al., 2001; Alemany et al., 2007). SphK1 was originally purified from rat and show a high degree of homology with the mouse and human enzyme (Kohama et al., 1998; Nava et al., 2000). The second isoform, SphK2, was cloned and characterized from mouse and human by Spiegel and colleagues (Liu H. et al., 2000). At the structural level, SphK1, in homology with SphK2, is composed of an N-terminal (NT) and a C-terminal (CT) domain, with the catalytic one located in a cleft at the interdomain junction. Both structures appear to have an homology because they contain five conserved domains, including an adenosine triphosphate (ATP)-binding motif that allows the transfer of a γ-phosphoryl group from the ATP to the D-*erythro*-sphingosine, to generate the S1P (Melendez et al., 2000; Nava et al., 2000). However, while SphK2 presents a nuclear localization signal (NLS) in the NT and a nuclear exportation signal (NES) in the CT, SphK1 lacks these sequences. Importantly, their presence within the SphK2 structure increases also the number of amino acids required for the enzyme composition. In fact, SphK1 consists of 384 amino acids (42.5 kDa), whereas SphK2 presents with 618 amino acids (65.2 kDa) (Liu H. et al., 2000; Okada et al., 2005). Moreover, based on kinetic studies performed *in vitro* and *in vivo*, both SphKs cannot only phosphorylate sphingosine in a similar manner, but they can also phosphorylate the immunomodulatory drug FTY720 (Fingolimod). However, SphK2 appear to be more efficient in doing so than SphK1 (Billich et al., 2003; Paugh et al., 2003). Due to this property, it has been suggested that only SphK2 is required for metabolic activation of this drug. In fact, FTY720, that is able to cause lymphopenia, loses its effect only in mice lacking SphK2, but not in mice in which SphK1 is downregulated (Allende et al., 2004; Kharel et al., 2005). For all these very reasons, it appears crystal-clear that, based on their structure, both the kinases can have different functions and localization (please, see more below). However, as shown by previous studies, these kinases have overlapping vital functions. In this regard, although knockout mouse models for either SphK1 or SphK2 develop normally, the genetic deletion of both isoforms results in fetal death due to alterations in vasculogenesis and severe bleeding (Allende et al., 2004; Mizugishi et al., 2005; Michaud et al., 2006).

### Subcellular Localization of SphKs

Concerning the localization of the two enzymes, it has been reported that SphK1 predominantly resides in the cytoplasm (Wattenberg, 2010; Pitson, 2011), and it can translocate to the plasma membrane upon cell stimulation (Pitson et al., 2003). Therefore, S1P generated by SphK1 can be exported outside the cells, and activate both proliferative and anti-apoptotic effects, in an autocrine and/or paracrine manner (Spiegel and Milstien, 2000). This phenomenon is known as “inside-out” signaling, and it has been described in cardiac myocytes too (Tao et al., 2007; Wang et al., 2012). Moreover, Ancellin et al. (2002) demonstrated that SphK1 can be released outside the cells, thus accounting for the extracellular S1P generation. Conversely, SphK2 is primarily localized at the level of the endoplasmic reticulum (ER, Maceyka et al., 2005), or it can also be associated with mitochondria (Strub et al., 2011; Chipuk et al., 2012). S1P generated in these compartments can affect cell survival (Maceyka et al., 2005; Strub et al., 2011; Chipuk et al., 2012; Maceyka et al., 2012). Moreover, since SphK2 presents an NLS and NES, it can shuttle in and out of the nucleus. S1P generated in this subcellular compartment can affect histone deacetylases activity, with a consequently enhanced transcription of genes involved in the growth arrest (Hait et al., 2009).

### Functional Role of SphK1

As we learned before, it is generally well-consolidated that SphK1 is a cell survival promoter (Karliner, 2013). Elevated cellular SphK1 levels appear to play a major role in enhanced proliferation and metastasis/invasion of several types of cancer cells (Xia et al., 2000; Johnson et al., 2005; Li et al., 2008; Shida et al., 2008; Pyne et al., 2016). In this context, more than one study has demonstrated that inhibition of SphK1 has considerable potential as an anti-cancer strategy (Shida et al., 2008; Pyne et al., 2016). Similarly, the downregulation of SphK1 has proven able to induce apoptosis and confer sensitivity to chemo- or radiation therapy of cancer cell lines (Baran et al., 2007; Shida et al., 2008; Guillermet-Guibert et al., 2009; Pyne et al., 2016). In line with these reports, ventricular cardiomyocytes and cardiac fibroblasts lacking SphK1 exhibit greater cell death when subjected to hypoxia, compared to wild-type (WT) controls (Ancellin et al., 2002; Tao et al., 2007). Interestingly, treatment of cardiomyocytes with exogenous S1P or with monoganglioside (GM-1), an acidic glycosphingolipid containing one sialic acid residue shown to elicit S1P generation (Cavallini et al., 1999), enhances the survival of both WT and SphK1 null cells (Ancellin et al., 2002; Tao et al., 2007). It is worth stressing that one of the proposed mechanism by which SphK1 controls cell death is the regulation of ceramide (Maceyka et al., 2005). In contrast to S1P, the ceramide-signaling molecule is able to exert pro-apoptotic actions. Its synthesis and accumulation are enhanced in cells lacking SphK1, while prevented in presence of high SphK1 levels (Maceyka et al., 2005). In 1996, in order to tie together the ability of S1P and ceramide to control cell fate, it has been coined the term “sphingolipid rheostat” (Cuvillier et al., 1996; Newton et al., 2015). However, although SphK1 appears to play a major role in the regulation of this “rheostat,” previous studies suggested that sphingolipid *per se* are able to influence the whole mechanism, thus including the “inside-out” one. In this context, Huang et al. (2014) have recently shown that S1P can activate a positive feedback amplification loop via S1PRs activation and consequent increase in SphK1 expression.

### Functional Role of SphK2

Opposite to the protective role attributed to SphK1, several early studies examining SphK2’s role have documented that the overexpression of this kinase induces cell cycle arrest and apoptosis (Karliner, 2013). More in detail, SphK2 can inhibit cell growth and enhance apoptosis, in part by increasing ceramide production (Maceyka et al., 2005). Similarly, mitochondrial localization of SphK2, and specifically S1P generation at this site seems to contribute to the activation of the pro-death Bcl-2 family protein, BID, with subsequent mitochondrial membrane permeabilization and cytochrome *c* release (Strub et al., 2011; Chipuk et al., 2012). In keeping with this view, several reports have shown that downregulating SphK2 can effectively prevent the increase in apoptotic rates induced by the administration of either TNF-α or staurosporine (Weigert et al., 2007; Hofmann et al., 2008). Interestingly, and contrary to the dogma that, differently from Sphka1, SphK2 is a pro-death factor, recent experimental evidence now supports a key role for this kinase in promoting cell survival and proliferation, much like SphK1 does in cancer cells, or even in cardiomyocytes (Weigert et al., 2009; Gomez et al., 2011; Vessey et al., 2011). Consonant to this view, Weigert et al. (2009) have reported that the genetic ablation of SphK2 in MCF-7 breast tumor xenografts results in the inhibition of tumor growth. Moreover, in isolated murine hearts, Karliner and colleagues have shown that SphK2 is necessary for successful ischemic pre- and post-conditioning (Vessey et al., 2011). Further to this, Gomez et al. (2011) also demonstrated that SphK2-evoked cardioprotection is dependent on the ability to prevent ischemia-induced mitochondrial dysfunction. Interestingly, the apparently divergent outcome reported with SphK2 studies could be ascribed to the specific subcellular localization of the enzyme. In agreement with this eventuality, studies have suggested that, when SphK2 is localized in the nucleus, it can inhibit the synthesis of DNA, thus exerting anti-proliferative effects (Hait et al., 2009). Conversely, other contributions have shown that, in human colon carcinoma cells, S1P generated by nuclear SphK2 can inhibit the retinoic acid receptor β, attenuating the tumor suppressor effects of this receptor (Shi et al., 2017).

## S1P: Generation and Function

### S1P Biosynthesis

**FIGURE 1:**
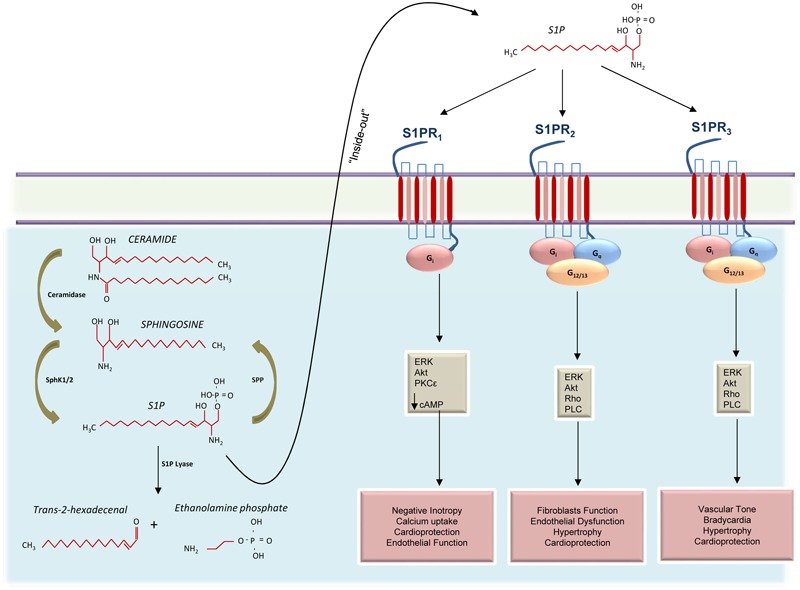
Schematic representation of S1PRs signaling activation in the cardiovascular system. Ceramide is catabolized by ceramidase to produce sphingosine that, in turn, is phosphorylated by sphingosine kinases 1 and 2 (SphK1 and 2) to generate the sphingosine 1-phosphate (S1P). Cellular S1P concentrations are regulated by the balance between its synthesis and de-phosphorylation mediated by S1P phosphatases (SPP). In the cytosol, S1P can also be irreversibly cleaved into *trans*-2-hexadecenal and ethanolamine phosphate or can be exported out of cells through the “inside-out” mechanism where it can bind three different receptors (S1PR_1-3_). Activation of S1PR_1_ induces negative inotropic effects via G protein (Gi) activation and decreased cAMP concentration. Moreover, S1PR_1_ is able to positively affect endothelial function and to confer protection to the heart, via activation of the mitogen-activated protein kinase 1 and 2 (ERK) and the protein kinase B (Akt). Both S1PR_2_ and S1PR_3_ activate Gi, Gq, and G12/13 appear to collaborate in providing cardioprotection and in regulating cardiac hypertrophy. However, while S1PR_2_ is involved in endothelial dysfunction and promotion of fibroblasts function, S1PR_3_ appears to influence the vascular tone and induce bradycardia.

Given the variety of processes involving S1P, most cells—red blood cells, platelets, and vascular ECs, in particular—have all the enzymatic machinery necessary for S1P synthesis. Like other sphingolipids, S1P is derived from ceramide which is composed of a sphingosine base and an amide-linked acyl chain of variable length (Yu and Law, 2009). Ceramide is in turn produced from the *de novo* synthetic pathway initiated by serine palmitoyltransferase in the ER, or from the degradation of some sphingolipids (Dolgachev et al., 2004; Reynolds et al., 2004). The intracellular deacylation by ceramidase gives way to the formation of sphingosine and carboxylate (Park and Schuchman, 2006). Then sphingosine can be phosphorylated to produce S1P (**Figure 1**). However, it is worth recalling that S1P levels in the cell are regulated not only by S1P biosynthetic enzymes but also by S1P degradative pathways, such as SPLs—two S1P-specific phosphatases—and by three lipid phosphate phosphatases (**Figure 1**; Hannun et al., 2001).

### S1P Functions

In general, S1P cell death-suppressing and cell survival promoting effects are opposite to those typically attributed to ceramide that, almost invariably, induces apoptosis, senescence, autophagy, and growth arrest (Cuvillier et al., 1996; Zheng et al., 2006; Pruett et al., 2008; Saddoughi and Ogretmen, 2013). Therefore, the intracellular synthesis of S1P vs. ceramide should be always under a tight control because any subtle change in this finely tuned balance, in response to environmental changes and stimuli, can direct cell in one direction or in the other (Cuvillier et al., 1996; Zheng et al., 2006; Pruett et al., 2008; Saddoughi and Ogretmen, 2013). Along with this, SPL is able to promote apoptosis under stress conditions, reducing the circulating levels of S1P (Kumar et al., 2011). Importantly, Karliner and colleagues have demonstrated that SPL activation in the myocardium following ischemia leads to reduced S1P levels and that knockout mice for this enzyme exhibit higher S1P levels and smaller infarct size (Bandhuvula et al., 2011). Moreover, mice null for SPL show significantly increased left ventricular function recovery over their WT infarcted counterparts (Bandhuvula et al., 2011). Thus, inhibition of SPL could represent a new target that can be utilized to prevent myocardium loss after ischemic injury.

### S1P Levels as a Biomarker of Cardiovascular Disease

In light of the evidence discussed above, it is important to consider that blood plasma and serum typically contains, in principle, high levels of S1P (Murata et al., 2000; Deutschman et al., 2003). Importantly, changes in serum S1P may be a predictive marker for the presence and severity of cardiovascular disease, as in the case of obstructive coronary artery disease (CAD), atherosclerosis, myocardial infarction (MI) and HF in humans (Deutschman et al., 2003; Sattler et al., 2010; Argraves et al., 2011; Cannavo et al., 2013b; Egom et al., 2013; Soltau et al., 2016).

Accordingly, Knapp et al. (2013) reported a reduction in circulating S1P levels in patients with acute MI as compared to controls. Similarly, we have recently demonstrated, in mouse and rat models of post-ischemic HF, that either cardiac or circulating levels of S1P are reduced compared to non-ischemic controls (Cannavo et al., 2013b, 2017).

However, it is important to consider that a large amount (∼60–80%) of S1P in the human plasma is associated with high-density lipoprotein (HDL). The importance of HDL is mainly due to the actions exerted by this lipoprotein on S1P function. In this context, Wilkerson et al. (2012) demonstrated that HDL-S1P are able to induce a lower rate of internalization and degradation of S1PR_1_ than albumin-S1P (another carrier of S1P). Of note, HDLs are usually reduced in several diseases like atherosclerosis, CAD, MI, renal insufficiency, and diabetes (Sattler et al., 2010), and this could influence the levels of circulating S1P. In the same vein, Sattler et al. (2010) reported that, in patients with CAD or MI, plasma S1P levels (normalized to HDL levels) were higher than in controls. However, in the same study, the authors analyzed the levels of HDL-S1P in MI and stable CAD patients, showing that the levels of S1P conjugated with this carrier were lower than in controls. In line with the latter evidence, Argraves et al. (2011) demonstrated that circulating S1P, dihydro-S1P and c24:1-ceramide levels in HDL correlate inversely with the incidence of ischemic heart disease.

## S1P Receptors and Dependent Signaling in Cardiovascular System

Many of S1P actions are mediated through specific GPCRs. Goodemote et al. (1995) demonstrated, for the first time, that S1P induced the activation of the extracellular signal-regulated kinase (ERK) 1/2 via Gi protein activation. Later on, Lee et al. (1998) identified a GPCR, originally termed endothelial differentiation gene 1 (EDG1), as the receptor of S1P. Currently, five closely related GPCRs (S1PR_1-5_), which differ in tissue and cell expression, have been identified for their high affinity for this bioactive lipid (Chun et al., 2002). Importantly, while S1PR_1-3_ are mostly expressed in the cardiovascular, central nervous system, and immune system, S1PR_4_ is mainly present in the lymphoid tissue, whereas S1PR_5_ is predominantly expressed in the central nervous system, immune system (natural killer cells), and spleen (Walzer et al., 2007; Pyne and Pyne, 2010; Jeffery et al., 2011). Importantly, the effects associated with S1PRs activation on different cell types in the cardiovascular system, either on cardiomyocytes, ECs, smooth muscle cells, or fibroblasts, are dictated by the specific G protein coupling. S1PR_1_ couples exclusively with the inhibitory G protein alpha subunit (Gαi), whereas S1PR_2_ and S1PR_3_ bind to Gαi, Gαq, and Gα13 and S1PR_4_ and S1PR_5_ couple to both Gαi and Gα13 (Means and Brown, 2009). Following ligand binding and subsequent activation, the α subunit of the heterotrimeric G protein is released and interacts with its downstream effectors. The main effector for Gαi is the adenylate cyclase, which is inhibited, thus leading to a reduction of the second messenger cyclic adenosine 3′,5′-monophosphate (cAMP) (Cannavo and Koch, 2017b). Moreover, Gαi is able to activate PKCα and 𝜀, thus modulating calcium uptake (Thompson et al., 2006; Marino et al., 2017). In contrast, phospholipase C (PLC) mediates the response due to Gαq activation. In turn, PLC hydrolyzes phosphatidylinositol 4,5-bisphosphate (PIP2) to DAG and inositol trisphosphate (IP3) (Cannavo and Koch, 2017b), whereas that for Gα13 and Gα12 is a Rho guanine nucleotide exchange factor (Rho-GEF), activating downstream low molecular Rho GTPases (Sugimoto et al., 2003).

### S1PRs in Vasculature

S1P plays a key role in the development of the vasculature and its stabilization (Schuchardt et al., 2011; Cannavo et al., 2013b). Accordingly, S1P regulates the growth of ECs and vascular smooth muscle cells (VSMCs) (Skoura and Hla, 2009; Schuchardt et al., 2011; Kerage et al., 2014). ECs express S1PR_1_, S1PR_2_, and S1PR_3_, with the isoform 1 being the most expressed subtype (Skoura and Hla, 2009; Kerage et al., 2014). For these reasons, it is not surprising that most of the S1P-mediated responses in these cells occur via S1PR_1_. Importantly, S1P regulates and stimulates the migration and proliferation of ECs and alteration in S1P levels or activity are responsible for aberrant vascular maturation (Kerage et al., 2014). It has been shown, for instance, that the inability of S1P to activate the GTPase Rac, as observed in global S1PR_1_ knockout mice, is one of the mechanisms associated with the impaired vasculature development and embryonic lethality (Liu Y. et al., 2000). At this regard, in this study, the authors suggest that the alteration of S1PR_1_ activation on ECs could negatively affect VSMCs recruitment (Liu Y. et al., 2000). Importantly, in addition to S1PR_1_ binding in ECs, S1P also activates the S1PR_3_ eliciting important vascular processes, such as the formation of new vessels and stabilization of barrier integrity (Waeber et al., 2004). For example, it has been shown that S1P-mediated migration proliferation and vasculature formation require both the S1PR_1_ mediated activation of Gi protein and the S1PR_3_ coupling to Gq/G12,13 (Sugimoto et al., 2003; Kono et al., 2004; Waeber et al., 2004). The S1PR_3_ requirement has been further validated in studies demonstrating that a peptide derived from the second intracellular loop of the S1PR_3_ can induce pro-angiogenic responses (Licht et al., 2003). Conversely, the activation and upregulation of S1PR_2_ has been associated with impaired functions in ECs, i.e., chemotactic, wound healing, and morphogenic responses (Lu et al., 2012; Zhao et al., 2015). In particular, Lu et al. (2012) have recently demonstrated *in vivo* that, in aging rats, S1PR_2_, like others GPCRs (Vasto et al., 2010; Ferrara et al., 2014), takes part in senescence-mediated endothelial dysfunction and aging processes (Zhao et al., 2015).

Interestingly, in VSMCs the expression pattern for S1PRs significantly differs from that of ECs (Waeber et al., 2004; Kerage et al., 2014). VSMCs mainly express the S1PR_2_ and S1PR_3_ (Waeber et al., 2004; Kerage et al., 2014). While S1PR_3_ stimulation increases the activity of Rac with an increased VSMCs migratory capacity, the activation of S1PR_2_ inhibits Rac via Rho/Rho kinase pathway, thus leading to a significant reduction in VSMCs function (Waeber et al., 2004; Szczepaniak et al., 2010; Kerage et al., 2014). Importantly, S1PR_3_ appears to be the major mediator of S1P-induced vasoconstriction because this lipid fails to increase the vascular tone in arteries isolated from S1PR_3_ knockout mice (Salomone et al., 2008).

### S1PRs in the Heart

Myocytes and fibroblasts represent the vast majority of the myocardial tissue. Importantly, due to the elevated activity expression of SphKs, fibroblasts appear to be the major source of cardiac S1P (Kacimi et al., 2007). Cardiac fibroblasts express predominantly S1PR_3_, with much lower levels of S1PR_1_ and S1PR_2_ being expressed (Landeen et al., 2008). However, most of the effects on fibroblasts function have been attributed to S1PR_2_ activation (Wang et al., 1997; Olivera et al., 1999; Urata et al., 2005). In particular, S1P is a positive regulator of fibroblasts function and proliferation, and these effects are associated with the activation of ERK and Rho activity downstream of S1PR_2_ (Wang et al., 1997; Olivera et al., 1999; Urata et al., 2005). Differently from fibroblasts, in cardiomyocytes, S1PR_1_ is the predominant S1P receptor subtype expressed (Means et al., 2008; Cannavo et al., 2013b). Initially, several studies evaluated the effect on S1PR_1_ on ion channels and contractility (Means and Brown, 2009), but decades of discoveries have later revealed that this receptor has also a prominent role in hypertrophic response and cardioprotection (Means and Brown, 2009).

S1P-dependent activation of S1PR_1_ in cardiomyocytes is necessary for heart development in mice (Clay et al., 2016). In fact, Clay et al. (2016) have recently shown that conditional knockout mice for S1PR_1_ show ventricular septal defects and perinatal lethality. Moreover, these authors reported that lacking S1PR_1_ is associated with decreased myofibril organization (Clay et al., 2016). Similarly, Keul et al. (2016) have shown that cardiomyocyte-restricted deletion of S1PR_1_ in mice results in progressive cardiomyopathy, compromised response to β-adrenergic receptor (βAR) stimulation and premature death.

The S1PR_1_ in cardiomyocytes is also a major regulator of contractile response (Means and Brown, 2009). Indeed, it counters the mechanical effects (positive inotropy/lusitropy, etc.) that follow cardiac β_1_-adrenergic receptor (β_1_AR)-agonism (Means and Brown, 2009; Cannavo et al., 2013b). Accordingly, S1P treatment of ventricular myocytes blocks the effects produced by the β_1_/β_2_AR agonist, isoproterenol, preventing the activation of adenylate cyclase, thus leading to a negative inotropic response (Means et al., 2008). This functional interaction between βAR and S1PR_1_ signaling *in vivo* was more recently reported also by Errami et al. (2008). In their study, these authors demonstrated that βAR-agonism in mice results in cardiac hypertrophic response via engagement of the S1PR_1_ signaling pathway (Errami et al., 2008). We recently proposed that this cross-talk is a major protective mechanism in response to myocardial ischemia (Cannavo et al., 2013b). In fact, we showed that isoproterenol stimulation of H9c2 cells induces the activation of S1PR_1_ pro-hypertrophic signaling (Cannavo et al., 2013b). *In vivo*, we observed that, following a cardiac ischemic attack, increased circulating levels of catecholamines lead to β_1_AR hyperactivation and subsequent desensitization/downregulation (Cannavo et al., 2013b), an effect coupled to increased GPCR kinase 2 (GRK2) levels that regulate both the β_1_AR and S1PR_1_ (Cannavo et al., 2013b). Importantly, the activation of such signaling pathway leads to the reciprocal downregulation of β_1_AR and S1PR_1_ in cardiac myocytes, leading to worse remodeling and progression toward HF (Cannavo et al., 2013b). This study further supported the idea that blockade of GRK2 is a valid strategy to prevent HF development and progression, but also demonstrated the cardioprotective role of S1PR_1_ (Cannavo et al., 2013a, 2016a,b; Cannavo and Koch, 2017a). In line with these data, we recently reported that activation of S1PR_1_ in the heart is also modulated by the β_3_AR (Cannavo et al., 2017). This receptor is the less βAR isoform expressed in the heart; however, it has important regulatory activities in cardiac hypertrophic response and in contractility (Cannavo and Koch, 2017b). In line with a previous study in adipocytes (Zhang et al., 2014), we demonstrated that selective β_3_AR stimulation leads to SphK1 upregulation and S1P release with a subsequent activation of S1PR_1_ in cardiomyocytes (Cannavo et al., 2017). Again, this mechanism appears to be relevant in a post-ischemic HF animal model. In fact, re-activation of β_3_AR via β1-AR blockade (Metoprolol), an indirect agonist of β_3_AR, is able to promote the activation of S1PR_1_ thus protecting the heart from failure (Cannavo et al., 2017).

Importantly, as discussed above, S1P is a cardioprotective molecule which signals in the heart via S1PRs. However, despite decades of studies from several groups, including ours, suggesting that S1PR_1_ is the major player in S1P-dependent cardioprotection (Cannavo et al., 2013a, 2017; Karliner, 2013; Marino et al., 2017), some reports have indicated that S1PR_2_ and S1PR_3_ can also take part in these protective molecular mechanisms activated in the myocardium in response to a specific injury (Theilmeier et al., 2006; Means et al., 2007; Means and Brown, 2009; Morel et al., 2016; Ruiz et al., 2017; Yung et al., 2017). In particular, mice lacking either S1PR_2_ or S1PR_3_, following an ischemic insult, develop infarcts equivalent to those of WT mice, whereas in S1PR2 and 3 double-knockout mice, the infarct size was increased by more than 50%, thus suggesting the potential role of these two receptors in protecting cardiomyocytes (Means et al., 2007). Mechanistically, all the beneficial effects associated with S1PR_1-3_, appear to be dependent mainly on the protein kinase B (Akt), that, via augmentation of eNOS expression/activity, has multiple effects, such as induction of adaptive hypertrophy, modulation of angiogenesis, and inhibition of apoptosis (Mandala et al., 2002; Means and Brown, 2009; Chaanine and Hajjar, 2011; Cannavo et al., 2013c; Keul et al., 2016). Moreover, it is also worth noting that recent reports suggest a protective role for RhoA activation in the heart (Xiang et al., 2013; Zhao et al., 2014; Yung et al., 2017) which do not involve S1PR_1_ (**Figure 1**).

## Targeting S1Prs and SphKs as a Therapeutic Strategy in Cardiovascular Disorders

Currently, the majority of drugs targeting the S1P signaling are directed to the S1PRs rather than the ligand. This is due to the fact that, as anticipated above, almost all S1P actions are mediated by its receptor. Moreover, targeting a specific S1PR would render a given drug highly selective. For these reasons, many agonists/antagonists of the S1PRs have been developed and studied. For some of them, clinical data on are also available (Amiselimod, Siponimod, Ozanimod, Ceralifimod, GSK2018682, Ponesimod; O’Sullivan and Dev, 2017; Sugahara et al., 2017). One of the most tested S1PR agonists is Fingolimod or FTY720. This compound is a structural homolog of S1P, which is phosphorylated by SphK2 to form Fingolimod-phosphate (Fingolimod). It serves as a potent agonist of S1PR_1_ (Mandala et al., 2002; Hla and Brinkmann, 2011), or as a SphK1 inhibitor (Tonelli et al., 2010). Of relevance, this compound is a US Food and Drug Administration (FDA) approved the drug for the treatment of multiple sclerosis (Jeffery et al., 2011; Pelletier and Hafler, 2012). In fact, although the Fingolimod-phosphate initially activates S1PR_1_, on lymphocytes, it subsequently can induce the receptor downregulation thus preventing the egress of these cells from lymphoid tissues (Mandala et al., 2002; Jeffery et al., 2011; Pelletier and Hafler, 2012). This double mechanism of action (agonism/antagonism) of Fingolimod on S1PR_1_ reduces the infiltration of lymphocytes into the central nervous system blocking their noxious effect (Mandala et al., 2002; Jeffery et al., 2011; Pelletier and Hafler, 2012).

## S1P and Protection Against Myocardial Ischemia

S1P is formed in the ischemic myocardium, and it is thought to be cardioprotective, mimicking the effects of ischemic preconditioning via a PKC𝜀-dependent pathway (Jin et al., 2004; Vessey et al., 2009; Marino et al., 2017). Importantly, as suggested by several studies in cardiac and non-cardiac cells, these beneficial effects are induced mainly by the selective binding to S1PR_1_. In line with this possibility, studies have evaluated the potential protective effect of S1PR_1_ agonism in cardiac cells. For instance, Wang et al. (2014) demonstrated that Fingolimod increases survival in adult murine cardiac myocytes subjected to hypoxia by inhibiting apoptosis. Following *in vivo* studies have demonstrated cardioprotective effects exerted by S1PR_1_ via Fingolimod stimulation. In particular, reports have shown that this drug is able to reduce ischemia/reperfusion (I/R) injury and to improve myocardial function in isolated mouse and rat heart preparations (Hofmann et al., 2009; Egom et al., 2010; Vessey et al., 2013). Further to this, in a mouse model of myocardial I/R, Goltz et al. (2015) have shown that, when given at reperfusion, Fingolimod provides a better hemodynamic outcome as compared to placebo-treated animals. Importantly, this effect was associated with a reduction in the number of phagocytic monocytes invading the myocardium (Goltz et al., 2015). Furthermore, in a recent report, Santos-Gallego et al. (2016) have demonstrated that Fingolimod improves myocardial function after MI in pigs. In this study, the authors showed that Fingolimod administration was associated with a reduction in the cardiac hypertrophic response and interstitial fibrosis in the remote, non-ischemic myocardium (Santos-Gallego et al., 2016). However, it is worth noting that in addition to S1PR_1_ activity modulation, studies have demonstrated that, albeit with minor affinity, Fingolimod can also bind and activate S1PR_3_ (Mandala et al., 2002; Hla and Brinkmann, 2011; Karliner, 2013). For this reason, in light of this evidence, one question would be whether or not Fingolimod induces its effects and if these are really due to the only S1PR_1_ modulation or if they are also related to S1PR_3_. Answering this intriguing would require fully dedicated studies. Anyway, other reports concerning the S1PR_1_ agonism protective effects against ischemic injury have explored the role of these receptors also in other organs, such as the brain and the lung (Stone et al., 2015; Brait et al., 2016). In particular, Stone et al. (2015) have demonstrated that selective stimulation of S1PR_1_, via Fingolimod or VPC01091, provided comparable protection from a lung injury and dysfunction after I/R. Further, in a mouse model of stroke, Brait et al. (2016) demonstrated the potential of S1PR_1_ agonists, such as LASW1238 or Fingolimod, in reducing the infarct size. Importantly, the authors concluded that these two drugs protect the brain only when lymphopenia is sustained for at least 24 h (Brait et al., 2016). In aggregate, these studies strongly support the overall notion that pharmacological activation of S1PR_1_ can reduce the detrimental effects of acute ischemia in an experimental setting, as demonstrated both in small and in large-animal models.

### Use of S1PR_1_ Agonism in Humans: Any Alternative to Fingolimod?

**FIGURE 2:**
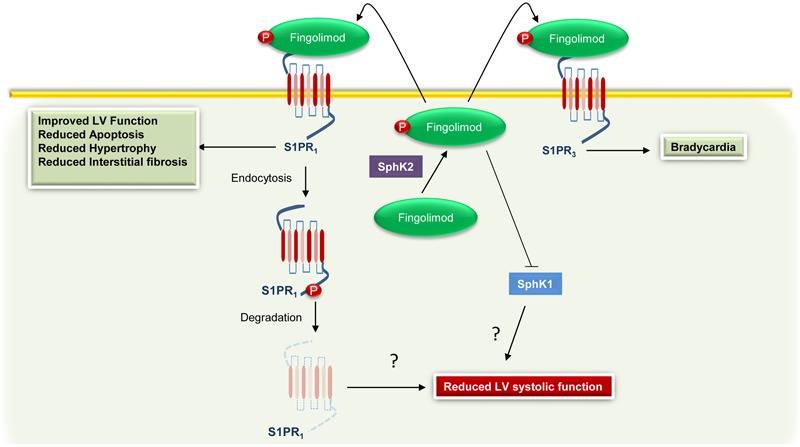
Cardiac effects associated with Fingolimod-mediated activation of S1PR_1_ and S1PR_3_. Fingolimod is phosphorylated by sphingosine kinase 2 (SphK2) to generate Fingolimod-phosphate (Fingolimod) that in turn binds to S1PR_1_. However, the sustained stimulation leads to S1PR_1_ internalization and degradation and to sphingosine kinase 1 (SphK1) inhibition. These effects are probably related to an impairment of the function of the left ventricle (LV). Moreover, via binding and activation of S1PR_3_, Fingolimod can induce bradycardia.

Although promising, the utilization of Fingolimod in humans raises some concerns. For instance, Sanna et al. (2004) demonstrated that stimulation with a non-selective S1PR agonist reduced heart rate (bradycardia) in WT mice, but not in S1PR_3_ KO animals. Moreover, Fingolimod is a well-known activator of both S1PR_1_ and S1PR_3_; hence, it has been suggested that behind direct S1PR_1_ activation, Fingolimod can also induce bradycardia via S1PR_3_ (Karliner, 2013). However, data obtained in rats by Fryer et al. (2012) show that Fingolimod-induced bradycardia requires only S1PR_1_. In line with this evidence, Gergely et al. (2012) showed that an S1PR_1_ selective ligand causes transient bradycardia in humans. Taking all this into account, the FDA revised the recommendations for cardiovascular monitoring in patients with multiple sclerosis receiving Fingolimod. Most importantly, in a recent study, Racca et al. (2016) demonstrated that in multiple sclerosis patients this pharmacological agent reduces left ventricular systolic function. Therefore, based on the beneficial effects exerted by S1PR_1_, it is plausible that this adverse event could be related to the antagonistic effect of the Fingolimod on S1PR_1_ (**Figure 2**). Therefore, other drugs or therapeutic strategies directed to improve S1P signaling/function are currently under evaluation. For instance, Sugahara et al. (2017) have recently tested the effects and the efficacy of Amiselimod, a second-generation S1P receptor modulator that is highly selective for S1PR_1_ and S1PR_5_, with no distinct agonist activity for S1PR_2_ or S1PR_3_. Importantly, compared to Fingolimod, this compound appears to be safer because it failed to induce bradycardia (Sugahara et al., 2017). In an alternative to approaches directed to modulate S1P signaling, Duan et al. (2007) have recently demonstrated that intracardiac injection of adenoviral vectors encoding for SphK1 markedly reduces myocardial infarct size, in a rat model of I/R injury. Of note, the overexpression of SphK1 results in a significant improvement in left ventricular systolic pressure and end-diastolic pressure, and better contractility (Duan et al., 2007). Alternatively, we recently demonstrated that restoration of cardiac plasma membrane levels of S1PR_1_, via a recombinant adeno-associated virus serotype 6 (AAV6), produces beneficial effects, in a HF rat model (Cannavo et al., 2013b). Importantly, the overexpression of S1PR_1_ improves both cardiac function and enhances the re-vascularization of the ischemic cardiac tissue (Cannavo et al., 2013b).

## Conclusion

The GPCRs are cell surface receptors that mediate fundamental processes in all cell types of the cardiovascular system. Therefore, it is not surprising that these receptors are currently the largest family of targets for drugs in clinical use. Accordingly, approximately 20% of medicaments used to treat cardiovascular disorders have GPCR-binding properties. Importantly, the discovery that S1P signals via GPCRs, influencing the entire mammal physiology—from the immune to the nervous system, from the circulation to the skeletal muscle apparatus—have significantly advanced the field of cardiovascular pharmacology. Several drug candidates, targeting both S1PRs and downstream molecules, are currently undergoing clinical trials, and novel compounds of diverse pharmacodynamics have been identified in the attempt to optimize the benefits afforded by S1PR_1_ stimulation during the course of acute and chronic cardiac diseases.

## Author Contributions

All authors listed have made a substantial, direct and intellectual contribution to the work, and approved it for publication.

## Conflict of Interest Statement

The authors declare that the research was conducted in the absence of any commercial or financial relationships that could be construed as a potential conflict of interest.
